# 3-Allyl-1-{[3-(4-nitro­phen­yl)-4,5-dihydro-1,3-oxazol-5-yl]meth­yl}-1*H*-anthra[1,2-*d*]imidazole-2,6,11(3*H*)-trione

**DOI:** 10.1107/S1600536811016606

**Published:** 2011-05-07

**Authors:** Zahra Afrakssou, Amal Haoudi, Frédéric Capet, Christian Rolando, Lahcen El Ammari

**Affiliations:** a Laboratoire de Chimie Organique Appliquée, Université Sidi Mohamed Ben Abdallah, Faculté des Sciences et Techniques, Route d’Immouzzer, BP 2202 Fès, Morocco; b Unité de Catalyse et de Chimie du Solide (UCCS), UMR 8181, Ecole Nationale Supérieure de Chimie de Lille, France; c USR 3290 Miniaturisation pour l’Analyse, la Synthèse et la Protéomique, 59655 Villeneuve d’Ascq Cedex, Université Lille-1, France; d Laboratoire de Chimie du Solide Appliquée, Faculté des Sciences, Université Mohammed V-Agdal, Avenue Ibn Battouta, BP 1014, Rabat, Morocco

## Abstract

The mol­ecular structure of the title compound, C_28_H_20_N_4_O_6_, consists of three fused six-membered rings (*A*,*B*,*C*) and one five-membered ring (*D*). The latter is linked to an isoxazole ring (*E*) *via* a methyl­ene unit. A 4-nitro-phenyl substituent (*F*) is attached to the isoxazole. The fused five and six-membered rings (*C*,*D*) are almost coplanar with an r.m.s. deviation of 0.0345 Å and make a dihedral angle of 9.40 (8)° with ring *A*. The isoxazole and 4-nitro-phenyl rings (*E*,*F*) are also almost coplanar with the imidazole and the fused adjacent ring (*C*,*D*), forming a dihedral angle of 11.4 (6)°. The crystal packing displays inter­molecular C—H⋯O hydrogen bonding. An intra­molecular C—H⋯O inter­action also occurs.

## Related literature

For the biological activity of anthraquinone derivatives, see: Agarwal *et al.* (2000 ▶); Barnard *et al.* (1995 ▶); Chen *et al.* (2007 ▶); Haug *et al.* (2003 ▶); Iizuka *et al.* (2004 ▶); Koyamaa *et al.* (2002 ▶); Su *et al.* (2005 ▶); Wu *et al.* (2005 ▶); Yen *et al.* (2000 ▶). For a derivative of the title compound, see: Afrakssou *et al.* (2010 ▶). For the use of related compounds as synthetic dyes, see: Simi *et al.* (1995 ▶). For puckering parameters, see: Cremer & Pople (1975 ▶).
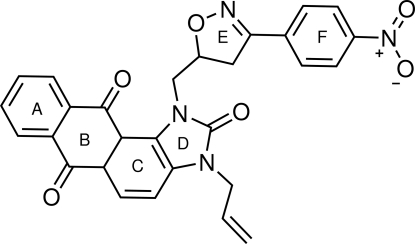


## Experimental

### 

#### Crystal data

C_28_H_20_N_4_O_6_
*M* *_r_* = 508.48
Monoclinic, 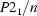

*a* = 10.0780 (3) Å
*b* = 22.7094 (6) Å
*c* = 11.2729 (3) Å
β = 113.809 (1)°
*V* = 2360.41 (11) Å^3^
*Z* = 4
Mo *K*α radiation
μ = 0.10 mm^−1^
*T* = 296 K
0.40 × 0.14 × 0.11 mm

#### Data collection

Bruker APEXII CCD diffractometer
Absorption correction: multi-scan (*SADABS*, Bruker, 2009) ▶ *T*_min_ = 0.704, *T*_max_ = 0.745
47772 measured reflections
4828 independent reflections
2998 reflections with *I* > 2σ(*I*)
*R*_int_ = 0.048

#### Refinement

*R*[*F* ^2^ > 2σ(*F* ^2^)] = 0.040
*wR*(*F* ^2^) = 0.111
*S* = 1.00
4828 reflections
343 parameters
H-atom parameters constrained
Δρ_max_ = 0.12 e Å^−3^
Δρ_min_ = −0.15 e Å^−3^

### 

Data collection: *APEX2* (Bruker, 2009 ▶); cell refinement: *SAINT-Plus* (Bruker, 2009 ▶); data reduction: *SAINT-Plus*; program(s) used to solve structure: *SHELXS97* (Sheldrick, 2008 ▶); program(s) used to refine structure: *SHELXL97* (Sheldrick, 2008 ▶); molecular graphics: *ORTEP-3 for Windows* (Farrugia, 1997 ▶); software used to prepare material for publication: *SHELXL97*.

## Supplementary Material

Crystal structure: contains datablocks I, global. DOI: 10.1107/S1600536811016606/im2283sup1.cif

Structure factors: contains datablocks I. DOI: 10.1107/S1600536811016606/im2283Isup2.hkl

Supplementary material file. DOI: 10.1107/S1600536811016606/im2283Isup3.cml

Additional supplementary materials:  crystallographic information; 3D view; checkCIF report

## Figures and Tables

**Table 1:** Hydrogen-bond geometry (Å, °)

*D*—H⋯*A*	*D*—H	H⋯*A*	*D*⋯*A*	*D*—H⋯*A*
C7—H7⋯O1^i^	0.93	2.60	3.516 (2)	169
C18—H18*B*⋯O2^ii^	0.97	2.37	3.333 (2)	170
C26—H26*B*⋯O1^i^	0.97	2.55	3.379 (3)	144
C16—H16*A*⋯O2	0.97	2.10	2.902 (2)	141

Symmetry codes: (i) 
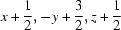
; (ii) 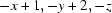.

**Fig. 1.**
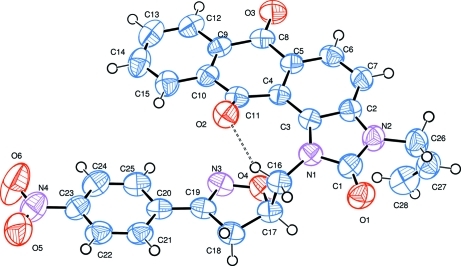
: Molecular plot of the title compound with the atom-labelling scheme. Displacement ellipsoids are drawn at the 50% probability level. H atoms are represented as small circles.

**Fig. 2.**
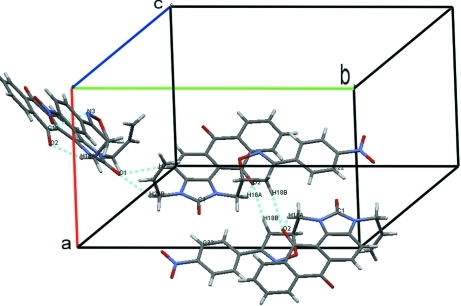
: Partial packing view showing the intermolecular C–H···O interactions.

## supplementary crystallographic information

### Comment

Recently, a number of pharmacological tests revealed that anthraquinone derivatives present various biological activities including antifungal (Agarwal *et al.*, 2000), antimicrobial (Wu *et al.*, 2005), anticancer (Koyamaa *et al.*, 2002; Su *et al.*, 2005; Chen *et al.*, 2007), antioxidant (Yen *et al.*, 2000; Iizuka *et al.*, 2004), and antihuman cytomegalovirus activity (Barnard *et al.*, 1995).

Aminoanthraquinone derivatives are a class of compounds largely used as phytotherapeutic drugs (laxatives, sedatives and antikidney and antibladder stones) and colouring agents (in the food, cosmetics and textile) (Simi *et al.*, 1995). Anthraquinone derivatives have also been utilized for the activation of human telomerase reverse transcriptase expression (Haug *et al.*, 2003), and they act as telomerase inhibitors or activators.

Due to their importance, in a previous study we have synthesized 1,3-diallyl-1*H*-anthra[1,2-*d*]imidazole-2,6,11(3*H*)-trione (Afrakssou *et al.*, 2010). Here we have focused on the reactivity of the exocyclic C=C bond of the allyl substituents towards nitriloxides. The latter are produced as intermediates in the dehydrohalogenation of 4-nitrobenzaldoxime by a solution of sodium hypochlorite. The oxime then reacts with 1,3-diallyl-1*H*-anthra[1,2-*d*] imidazole-2,6,11(3*H*)-trione in a biphasic medium (water-chloroform) at 0°C during 4 h to a unique cycloadduct 3-allyl-1-[3 -(4-nitro-phenyl)-4,5-dihydro-isoxazole-5-ylmethyl] -1,3-dihydro-anthra [1,2 d] imidazole-2,6,11-trione (Scheme 1). Due to this reaction sequence a racemate of the title compound which shows a stereogenic center at C(17) is formed.

Fig.1 shows the molecular plot of the crystal structure of the title compound. The isoxazole (E) adopts an envelope conformation on C(17) as indicated by the Cremer & Pople (1975) puckering parameters Q2 = 0.2117 (19) Å and φ2 = 141.6 (5)°. Moreover, ring (B) has a twisted conformation, with puckering parameters Q = 0.1480 (19) Å, θ = 114.6 (8) ° and φ = 139.7 (8) °, whereas all other rings are planar. The dihedral angles between fused five and six-membered rings (C,D) and the isoxazole ring (E) is 11.4 (6)°. The torsion angle between C27—C26—N2—C1 is 87.42 (0.22)°. In the crystal, adjacent molecules are linked by intermolecular C–H···O hydrogen bonding as shown in Fig. 2 and Table 1.

### Experimental

To a solution of 1,3-diallyl-1*H*-anthra[1,2-*d*] imidazole-2,6,11(3*H*)-trione (0.3 g, 0.87 mmol) and 4-nitrobenzaldoxime (0.36 g, 2.17 mmol) in chloroform (16 ml) was added dropwise a 24% sodium hypochlorite solution (8 ml) at 273 K. Stirring was continued for 4 h. The organic layer was dried over Na_2_SO_4_ and the solvent was evaporated under reduced pressure. The residue was then purified by column chromatography on silica gel using a mixture of hexane/ethyl acetate (1/1) as eluent. The title compound is formed as a racemate (Yield: 35%). Orange crystals are isolated after the solvent was allowed to evaporate.

### Refinement

All H atoms were located in a difference map and treated as riding with C—H = 0.93 Å for all aromatic H atoms, 0.97 Å for methylene and 0.98Å for methine with *U*_iso_(H) = 1.2 *U*_eq_ aromatic, methylene and methine. The C-bound H atoms of the allyl group were positioned geometrically and treated as riding with C—H = 0.93 Å (H27, H28A and H28B) and 0.97 Å (methylene) with *U*_iso_(H) = 1.2*U*_eq_.

The reflections (0 2 0) and (0 1 1) were omitted because the difference between their calculated and observed intensities are very large. They are affected by the beamstop.

### Crystal data

**Table:**

C_28_H_20_N_4_O_6_	*F*(000) = 1056
*M**_r_* = 508.48	*D*_x_ = 1.431 Mg m^−^^3^
Monoclinic, *P*2_1_/*n*	Melting point: 471 K
Hall symbol: -P 2yn	Mo *K*α radiation, λ = 0.71073 Å
*a* = 10.0780 (3) Å	Cell parameters from 7395 reflections
*b* = 22.7094 (6) Å	θ = 2.4–21.5°
*c* = 11.2729 (3) Å	µ = 0.10 mm^−^^1^
β = 113.809 (1)°	*T* = 296 K
*V* = 2360.41 (11) Å^3^	Prism, yellow
*Z* = 4	0.40 × 0.14 × 0.11 mm

### Data collection

**Table:**

Bruker APEXII CCD diffractometer	4828 independent reflections
Radiation source: fine-focus sealed tube	2998 reflections with *I* > 2σ(*I*)
graphite	*R*_int_ = 0.048
φ and ω scans	θ_max_ = 26.4°, θ_min_ = 2.5°
Absorption correction: multi-scan (*SADABS*, Bruker, 2009)	*h* = −12→12
*T*_min_ = 0.704, *T*_max_ = 0.745	*k* = −28→28
47772 measured reflections	*l* = −12→14

### Refinement

**Table:**

Refinement on *F*^2^	Primary atom site location: structure-invariant direct methods
Least-squares matrix: full	Secondary atom site location: difference Fourier map
*R*[*F*^2^ > 2σ(*F*^2^)] = 0.040	Hydrogen site location: inferred from neighbouring sites
*wR*(*F*^2^) = 0.111	H-atom parameters constrained
*S* = 1.00	*w* = 1/[σ^2^(*F*_o_^2^) + (0.0455*P*)^2^ + 0.4447*P*] where *P* = (*F*_o_^2^ + 2*F*_c_^2^)/3
4828 reflections	(Δ/σ)_max_ = 0.001
343 parameters	Δρ_max_ = 0.12 e Å^−^^3^
0 restraints	Δρ_min_ = −0.15 e Å^−^^3^

### Special details

**Table:**

Geometry. All s.u.'s (except the s.u. in the dihedral angle between two l.s. planes) are estimated using the full covariance matrix. The cell s.u.'s are taken into account individually in the estimation of s.u.'s in distances, angles and torsion angles; correlations between s.u.'s in cell parameters are only used when they are defined by crystal symmetry. An approximate (isotropic) treatment of cell s.u.'s is used for estimating s.u.'s involving l.s. planes.
Refinement. Refinement of *F*^2^ against ALL reflections. The weighted *R*-factor *wR* and goodness of fit *S* are based on *F*^2^, conventional *R*-factors *R* are based on *F*, with *F* set to zero for negative *F*^2^. The threshold expression of *F*^2^ > 2σ(*F*^2^) is used only for calculating *R*-factors(gt) *etc*. and is not relevant to the choice of reflections for refinement. *R*-factors based on *F*^2^ are statistically about twice as large as those based on *F*, and *R*- factors based on ALL data will be even larger.

### Fractional atomic coordinates and isotropic or equivalent isotropic displacement parameters (Å^2^)

**Table:**

	*x*	*y*	*z*	*U*_iso_*/*U*_eq_	
C1	0.6321 (2)	0.82375 (9)	0.19097 (19)	0.0612 (5)	
C2	0.85135 (19)	0.84194 (8)	0.35084 (17)	0.0552 (4)	
C3	0.81023 (17)	0.89317 (8)	0.27367 (15)	0.0502 (4)	
C4	0.90411 (17)	0.94182 (7)	0.30538 (15)	0.0475 (4)	
C5	1.04177 (18)	0.93398 (7)	0.40936 (15)	0.0505 (4)	
C6	1.0776 (2)	0.88249 (8)	0.48157 (16)	0.0593 (5)	
H6	1.1683	0.8794	0.5498	0.071*	
C7	0.9829 (2)	0.83582 (8)	0.45530 (17)	0.0622 (5)	
H7	1.0064	0.8018	0.5055	0.075*	
C8	1.1555 (2)	0.97997 (8)	0.44241 (17)	0.0574 (4)	
C9	1.1231 (2)	1.03401 (8)	0.36364 (17)	0.0556 (4)	
C10	0.98508 (19)	1.04342 (7)	0.26723 (16)	0.0527 (4)	
C11	0.86591 (19)	1.00068 (8)	0.24546 (16)	0.0524 (4)	
C12	1.2303 (2)	1.07627 (9)	0.3858 (2)	0.0712 (5)	
H12	1.3224	1.0703	0.4505	0.085*	
C13	1.2012 (3)	1.12668 (10)	0.3127 (2)	0.0793 (6)	
H13	1.2730	1.1550	0.3292	0.095*	
C14	1.0663 (3)	1.13554 (9)	0.2151 (2)	0.0754 (6)	
H14	1.0482	1.1692	0.1641	0.090*	
C15	0.9574 (2)	1.09440 (8)	0.19255 (19)	0.0651 (5)	
H15	0.8658	1.1008	0.1275	0.078*	
C16	0.59135 (18)	0.91052 (8)	0.05248 (16)	0.0576 (5)	
H16A	0.5982	0.9527	0.0669	0.069*	
H16B	0.4900	0.8995	0.0226	0.069*	
C17	0.64489 (18)	0.89559 (9)	−0.05152 (17)	0.0588 (5)	
H17	0.6223	0.8546	−0.0797	0.071*	
C18	0.58172 (18)	0.93738 (8)	−0.16582 (17)	0.0617 (5)	
H18A	0.5628	0.9177	−0.2474	0.074*	
H18B	0.4935	0.9560	−0.1695	0.074*	
C19	0.70447 (18)	0.98060 (8)	−0.13153 (16)	0.0553 (4)	
C20	0.69973 (18)	1.03873 (8)	−0.18912 (16)	0.0546 (4)	
C21	0.5711 (2)	1.06028 (9)	−0.28290 (18)	0.0640 (5)	
H21	0.4882	1.0370	−0.3120	0.077*	
C22	0.5655 (2)	1.11605 (9)	−0.33315 (19)	0.0701 (5)	
H22	0.4792	1.1305	−0.3955	0.084*	
C23	0.6886 (2)	1.15008 (8)	−0.2904 (2)	0.0662 (5)	
C24	0.8184 (2)	1.12962 (10)	−0.1991 (2)	0.0727 (6)	
H24	0.9011	1.1530	−0.1718	0.087*	
C25	0.8234 (2)	1.07410 (9)	−0.14903 (19)	0.0655 (5)	
H25	0.9106	1.0599	−0.0875	0.079*	
C26	0.7429 (2)	0.74050 (9)	0.3414 (2)	0.0749 (6)	
H26A	0.6442	0.7270	0.3176	0.090*	
H26B	0.7923	0.7385	0.4351	0.090*	
C27	0.8169 (2)	0.70030 (9)	0.2829 (2)	0.0776 (6)	
H27	0.8222	0.6608	0.3062	0.093*	
C28	0.8743 (3)	0.71473 (12)	0.2031 (3)	0.0922 (7)	
H28A	0.8718	0.7537	0.1768	0.111*	
H28B	0.9182	0.6862	0.1720	0.111*	
N1	0.67395 (14)	0.88059 (7)	0.17487 (13)	0.0554 (4)	
N2	0.73969 (16)	0.80148 (7)	0.30080 (15)	0.0633 (4)	
N3	0.82302 (15)	0.96221 (7)	−0.04143 (14)	0.0606 (4)	
N4	0.6801 (3)	1.20974 (9)	−0.3438 (2)	0.0907 (6)	
O1	0.52115 (15)	0.79872 (6)	0.12021 (14)	0.0768 (4)	
O2	0.73990 (14)	1.01619 (6)	0.18463 (13)	0.0677 (4)	
O3	1.27476 (15)	0.97262 (6)	0.53085 (13)	0.0835 (4)	
O4	0.80057 (12)	0.90603 (6)	0.00001 (12)	0.0663 (4)	
O5	0.5638 (2)	1.22624 (8)	−0.4262 (2)	0.1111 (6)	
O6	0.7864 (2)	1.24061 (9)	−0.3026 (3)	0.1445 (9)	

### Atomic displacement parameters (Å^2^)

**Table:**

	*U*^11^	*U*^22^	*U*^33^	*U*^12^	*U*^13^	*U*^23^
C1	0.0496 (11)	0.0657 (13)	0.0690 (12)	−0.0039 (10)	0.0245 (10)	−0.0025 (10)
C2	0.0513 (11)	0.0581 (11)	0.0572 (10)	−0.0025 (9)	0.0231 (9)	−0.0016 (8)
C3	0.0439 (10)	0.0582 (11)	0.0491 (9)	0.0041 (8)	0.0193 (8)	−0.0003 (8)
C4	0.0464 (10)	0.0516 (10)	0.0463 (9)	0.0034 (8)	0.0204 (8)	−0.0033 (7)
C5	0.0488 (10)	0.0559 (10)	0.0464 (9)	0.0008 (8)	0.0188 (8)	−0.0058 (8)
C6	0.0528 (11)	0.0668 (12)	0.0495 (9)	0.0057 (9)	0.0115 (8)	0.0018 (9)
C7	0.0623 (12)	0.0614 (12)	0.0578 (11)	0.0031 (10)	0.0190 (9)	0.0077 (9)
C8	0.0540 (11)	0.0654 (12)	0.0470 (9)	−0.0015 (9)	0.0145 (9)	−0.0099 (8)
C9	0.0584 (11)	0.0547 (11)	0.0557 (10)	−0.0037 (9)	0.0251 (9)	−0.0144 (8)
C10	0.0584 (11)	0.0489 (10)	0.0569 (10)	0.0057 (8)	0.0294 (9)	−0.0085 (8)
C11	0.0495 (11)	0.0581 (11)	0.0513 (9)	0.0077 (9)	0.0219 (8)	−0.0055 (8)
C12	0.0700 (13)	0.0670 (13)	0.0737 (13)	−0.0131 (11)	0.0258 (11)	−0.0153 (10)
C13	0.0819 (16)	0.0621 (14)	0.1010 (17)	−0.0139 (12)	0.0444 (14)	−0.0140 (12)
C14	0.0913 (17)	0.0502 (11)	0.0998 (16)	0.0048 (11)	0.0543 (14)	0.0004 (11)
C15	0.0718 (13)	0.0559 (11)	0.0748 (12)	0.0107 (10)	0.0371 (11)	−0.0013 (9)
C16	0.0391 (9)	0.0674 (11)	0.0611 (11)	0.0029 (8)	0.0147 (8)	0.0008 (9)
C17	0.0413 (10)	0.0693 (12)	0.0606 (10)	0.0026 (9)	0.0151 (8)	−0.0027 (9)
C18	0.0412 (10)	0.0799 (13)	0.0575 (10)	0.0015 (9)	0.0131 (8)	−0.0025 (9)
C19	0.0377 (10)	0.0742 (12)	0.0508 (9)	0.0038 (9)	0.0147 (8)	−0.0049 (9)
C20	0.0420 (10)	0.0692 (12)	0.0507 (9)	0.0026 (8)	0.0168 (8)	−0.0098 (8)
C21	0.0500 (11)	0.0681 (13)	0.0609 (11)	−0.0024 (9)	0.0090 (9)	−0.0084 (9)
C22	0.0600 (13)	0.0716 (13)	0.0649 (12)	0.0062 (11)	0.0109 (10)	−0.0085 (10)
C23	0.0684 (14)	0.0582 (12)	0.0739 (12)	0.0016 (10)	0.0309 (11)	−0.0113 (10)
C24	0.0553 (13)	0.0743 (14)	0.0889 (14)	−0.0077 (11)	0.0293 (11)	−0.0138 (12)
C25	0.0417 (10)	0.0818 (14)	0.0683 (12)	−0.0003 (10)	0.0174 (9)	−0.0063 (10)
C26	0.0668 (13)	0.0692 (13)	0.0855 (14)	−0.0114 (11)	0.0273 (11)	0.0125 (11)
C27	0.0647 (14)	0.0634 (13)	0.0915 (16)	−0.0019 (11)	0.0179 (12)	0.0047 (11)
C28	0.0823 (16)	0.0928 (17)	0.1015 (18)	−0.0009 (14)	0.0369 (15)	−0.0112 (14)
N1	0.0402 (8)	0.0646 (10)	0.0581 (8)	−0.0012 (7)	0.0164 (7)	0.0008 (7)
N2	0.0554 (10)	0.0604 (10)	0.0703 (10)	−0.0051 (8)	0.0216 (8)	0.0070 (8)
N3	0.0408 (8)	0.0796 (11)	0.0598 (9)	0.0054 (8)	0.0188 (7)	0.0010 (8)
N4	0.0901 (16)	0.0661 (13)	0.1166 (17)	0.0044 (12)	0.0426 (14)	−0.0096 (12)
O1	0.0551 (8)	0.0801 (10)	0.0868 (9)	−0.0157 (7)	0.0197 (7)	−0.0064 (7)
O2	0.0528 (8)	0.0662 (8)	0.0803 (9)	0.0133 (6)	0.0230 (7)	0.0037 (6)
O3	0.0639 (9)	0.0907 (11)	0.0692 (8)	−0.0144 (8)	−0.0008 (7)	0.0015 (7)
O4	0.0420 (7)	0.0861 (10)	0.0672 (8)	0.0113 (6)	0.0182 (6)	0.0102 (7)
O5	0.1209 (16)	0.0787 (12)	0.1199 (14)	0.0141 (11)	0.0344 (13)	0.0101 (10)
O6	0.1056 (16)	0.0775 (12)	0.233 (3)	−0.0174 (11)	0.0507 (16)	0.0068 (14)

### Geometric parameters (Å, °)

**Table:**

C1—O1	1.221 (2)	C16—H16B	0.9700
C1—N2	1.371 (2)	C17—O4	1.456 (2)
C1—N1	1.392 (2)	C17—C18	1.518 (2)
C2—C7	1.379 (2)	C17—H17	0.9800
C2—N2	1.384 (2)	C18—C19	1.502 (2)
C2—C3	1.411 (2)	C18—H18A	0.9700
C3—C4	1.404 (2)	C18—H18B	0.9700
C3—N1	1.405 (2)	C19—N3	1.286 (2)
C4—C5	1.420 (2)	C19—C20	1.463 (3)
C4—C11	1.477 (2)	C20—C21	1.390 (2)
C5—C6	1.387 (2)	C20—C25	1.396 (2)
C5—C8	1.483 (2)	C21—C22	1.379 (3)
C6—C7	1.376 (2)	C21—H21	0.9300
C6—H6	0.9300	C22—C23	1.373 (3)
C7—H7	0.9300	C22—H22	0.9300
C8—O3	1.224 (2)	C23—C24	1.378 (3)
C8—C9	1.472 (3)	C23—N4	1.471 (3)
C9—C12	1.390 (3)	C24—C25	1.374 (3)
C9—C10	1.393 (2)	C24—H24	0.9300
C10—C15	1.392 (2)	C25—H25	0.9300
C10—C11	1.486 (2)	C26—N2	1.455 (2)
C11—O2	1.2264 (19)	C26—C27	1.490 (3)
C12—C13	1.371 (3)	C26—H26A	0.9700
C12—H12	0.9300	C26—H26B	0.9700
C13—C14	1.375 (3)	C27—C28	1.294 (3)
C13—H13	0.9300	C27—H27	0.9300
C14—C15	1.384 (3)	C28—H28A	0.9300
C14—H14	0.9300	C28—H28B	0.9300
C15—H15	0.9300	N3—O4	1.408 (2)
C16—N1	1.460 (2)	N4—O6	1.206 (3)
C16—C17	1.514 (2)	N4—O5	1.224 (2)
C16—H16A	0.9700		
			
O1—C1—N2	126.88 (18)	O4—C17—H17	110.8
O1—C1—N1	126.32 (18)	C16—C17—H17	110.8
N2—C1—N1	106.80 (16)	C18—C17—H17	110.8
C7—C2—N2	128.61 (17)	C19—C18—C17	99.82 (14)
C7—C2—C3	123.44 (17)	C19—C18—H18A	111.8
N2—C2—C3	107.93 (15)	C17—C18—H18A	111.8
C4—C3—N1	134.81 (15)	C19—C18—H18B	111.8
C4—C3—C2	119.47 (15)	C17—C18—H18B	111.8
N1—C3—C2	105.71 (15)	H18A—C18—H18B	109.5
C3—C4—C5	116.42 (15)	N3—C19—C20	119.80 (16)
C3—C4—C11	124.85 (15)	N3—C19—C18	113.39 (16)
C5—C4—C11	118.57 (15)	C20—C19—C18	126.82 (15)
C6—C5—C4	121.74 (16)	C21—C20—C25	118.73 (18)
C6—C5—C8	117.06 (16)	C21—C20—C19	120.55 (17)
C4—C5—C8	121.17 (15)	C25—C20—C19	120.70 (16)
C7—C6—C5	121.98 (16)	C22—C21—C20	120.41 (18)
C7—C6—H6	119.0	C22—C21—H21	119.8
C5—C6—H6	119.0	C20—C21—H21	119.8
C6—C7—C2	116.76 (17)	C23—C22—C21	119.46 (19)
C6—C7—H7	121.6	C23—C22—H22	120.3
C2—C7—H7	121.6	C21—C22—H22	120.3
O3—C8—C9	120.70 (17)	C22—C23—C24	121.52 (19)
O3—C8—C5	120.92 (17)	C22—C23—N4	118.7 (2)
C9—C8—C5	118.36 (16)	C24—C23—N4	119.8 (2)
C12—C9—C10	119.54 (18)	C25—C24—C23	118.87 (19)
C12—C9—C8	120.01 (17)	C25—C24—H24	120.6
C10—C9—C8	120.45 (16)	C23—C24—H24	120.6
C15—C10—C9	119.50 (17)	C24—C25—C20	120.99 (18)
C15—C10—C11	119.50 (17)	C24—C25—H25	119.5
C9—C10—C11	120.97 (16)	C20—C25—H25	119.5
O2—C11—C4	122.39 (16)	N2—C26—C27	113.34 (18)
O2—C11—C10	119.36 (16)	N2—C26—H26A	108.9
C4—C11—C10	118.15 (15)	C27—C26—H26A	108.9
C13—C12—C9	120.4 (2)	N2—C26—H26B	108.9
C13—C12—H12	119.8	C27—C26—H26B	108.9
C9—C12—H12	119.8	H26A—C26—H26B	107.7
C12—C13—C14	120.4 (2)	C28—C27—C26	126.6 (2)
C12—C13—H13	119.8	C28—C27—H27	116.7
C14—C13—H13	119.8	C26—C27—H27	116.7
C13—C14—C15	120.2 (2)	C27—C28—H28A	120.0
C13—C14—H14	119.9	C27—C28—H28B	120.0
C15—C14—H14	119.9	H28A—C28—H28B	120.0
C14—C15—C10	120.0 (2)	C1—N1—C3	109.62 (14)
C14—C15—H15	120.0	C1—N1—C16	117.89 (14)
C10—C15—H15	120.0	C3—N1—C16	131.04 (15)
N1—C16—C17	112.46 (14)	C1—N2—C2	109.85 (15)
N1—C16—H16A	109.1	C1—N2—C26	122.97 (16)
C17—C16—H16A	109.1	C2—N2—C26	126.32 (16)
N1—C16—H16B	109.1	C19—N3—O4	109.50 (14)
C17—C16—H16B	109.1	O6—N4—O5	123.0 (2)
H16A—C16—H16B	107.8	O6—N4—C23	118.8 (2)
O4—C17—C16	108.55 (14)	O5—N4—C23	118.2 (2)
O4—C17—C18	104.60 (14)	N3—O4—C17	107.87 (12)
C16—C17—C18	111.04 (15)		

### Hydrogen-bond geometry (Å, °)

**Table:**

*D*—H···*A*	*D*—H	H···*A*	*D*···*A*	*D*—H···*A*
C7—H7···O1^i^	0.93	2.60	3.516 (2)	169
C18—H18B···O2^ii^	0.97	2.37	3.333 (2)	170
C26—H26B···O1^i^	0.97	2.55	3.379 (3)	144
C16—H16A···O2	0.97	2.10	2.902 (2)	141

Symmetry codes: (i) *x*+1/2, −*y*+3/2, *z*+1/2; (ii) −*x*+1, −*y*+2, −*z*.
